# Effects of inactivated *Lactobacillus rhamnosus* on growth performance, serum indicators, and colonic microbiota and metabolism of weaned piglets

**DOI:** 10.1186/s12917-024-04133-5

**Published:** 2024-09-20

**Authors:** Zhiheng Shu, Junhao Zhang, Qingwen Zhou, Yingjie Peng, Yuanhao Huang, Yi Zhou, Jun Zheng, Manya Zhao, Chao Hu, Shile Lan

**Affiliations:** 1 College of Bioscience and Biotechnology, Hunan Agricultural University, Changsha, 410128 China; 2 Guangdong Chuangzhan Bona Agricultural Technology Co., Ltd, Guangning, 526339 China

**Keywords:** Weaned piglets, Inactivated *lactobacillus rhamnosus*, Colonic microbiota, Metabolome

## Abstract

**Background:**

To assess the effects of inactivated *Lactobacillus rhamnosus* (ILR) on growth performance, serum biochemical indices, colonic microbiota, and metabolomics in weaned piglets, 120 piglets were randomly divided into five groups. Samples in the control group were fed a basal diet, while the experimental ILR1, ILR2, ILR3, and ILR4 groups were fed basal diets supplemented with 0.1%, 0.2%, 0.3%, and 0.4% ILR, respectively. The prefeeding period lasted for 5 days and was followed by a formal period of 28 days.

**Results:**

Compared to the control, the average daily gain increased by 4.38%, 7.98%, 19.32%, and 18.80% for ILR1, ILR2, ILR3, and ILR4, respectively, and the ratio of feed to gain decreased by 0.63%, 3.80%, 12.66%, and 10.76%, respectively. Serum IgA, IgG, IgM, total antioxidant capacity, and glutathione peroxidase levels increased significantly in weaned piglets in the treatment groups. Addition of 0.3% ILR significantly increased the Shannon and Simpson indices of the colonic microbiota in weaned piglets and altered the microbiota composition. Changes in metabolic profiles were observed and were primarily related to the urea cycle, amino acid metabolism, and lipid metabolism.

**Conclusion:**

ILR improved growth performance and serum immunological and biochemical indices and optimized the colonic microbiota structure and metabolism of weaned piglets.

## Background

The role of intestinal microbiota in host nutrition, growth, development, immunity, and health has been widely confirmed [1–3]. This extends the identification and functional study of probiotics in the gut microbiota [4, 5]. Common probiotics such as *Lactobacillus* and *Bifidobacterium* are typically consumed as active bacteria preparations [6]. *Lactobacillus* has been widely used in industry, medicine, and agriculture [7, 8]. Despite numerous reports supporting the health benefits of probiotics, the safety concerns resulting from the use of live bacteria remain controversial [9]. For example, horizontal gene transfer contributes to the spread of drug resistance in the gut microbiota [10–12]. Another important concern regarding the safety of live bacteria is the risk of translocation and subsequent bacteremia and septicemia [13]. Numerous cases of sepsis caused by *Lactobacillus rhamnosus* GG (LGG) have been reported clinically [14]. Probiotics may trigger an inflammatory response in highly susceptible individuals [15]. During breeding, the intestines of weaned piglets are weak, and the structure of the microbiota is incomplete, ultimately resulting in impaired antioxidant capacity and gastrointestinal function and leading to slow growth and even death [16]. Therefore, the addition of live bacteria to animal feed should be considered with caution. Furthermore, preservation conditions and quality control of live bacteria are difficult [17].

Inactivated lactic acid bacteria exhibit a beneficial nature similar to that of live bacteria [18, 19]. One study indicated that both live bacteria and heat-inactivated lactic acid bacteria reduced the aflatoxin content in PBS, and two inactivated *L. rhamnosus* (ILR) strains exhibited stronger adsorption capacities than did live bacteria [20]. Heat-inactivated lactic acid bacteria can release peptidoglycans, lipoteichoic acid, and extracellular polysaccharides (EPS) that all exert immunomodulatory effects [21]. Furthermore, heat-inactivated lactic acid bacteria still possess the ability to maintain the balance of intestinal microbiota [22, 23], and their products are easier to store and possess a longer expiration date [24].

*L. rhamnosus* regulates the gut microbiota and boosts immunity [25]. However, it has been reported that dietary supplementation with *L. rhamnosus* GG cannot prevent or reduce the adverse effects of *E. coli* F4 infection on the growth performance and health status of growing pigs, but it can decrease growth performance, increase diarrhea, and decrease serum immunoglobulin A (IgA) content [26]. Moreover, heat-killed *L. rhamnosus* improves growth performance and reduces diarrhea in growing pigs [27]. However, the effects of ILR on the structure and metabolism of the intestinal microbiota in weaned piglets have not been reported. Understanding the effects of ILR on the structure and metabolism of the intestinal microbiota in weaned piglets would help to systematically elucidate the mechanisms by which ILR promote growth and immunity. To provide a reference for the application of ILR in the production of weaned piglets, in this study the effects of ILR supplementation on growth performance, serum parameters, structure, and metabolism of the colonic microbiota in weaned piglets were analyzed.

## Methods

### Preparation of ILR

The *L. rhamnosus* strains used in this study were isolated from healthy pigs. Approximately 10.0 g of fresh fecal sample was weighed and transferred into a 250 mL sterile conical flask with 90 mL of sterile water and an appropriate amount of glass beads. After full shaking and mixing, 1 ml of mixed liquid was transferred to 100 mL of MRS medium and cultured at 37 °C for 48 h. After gradient dilution, 0.1 mL of the cultured medium was inoculated onto an MRS solid medium plate containing 1% CaCO_3_. After 48 h of culture at 37 °C, a single colony with obvious calcium lysosomes was picked and repeatedly purified on an MRS solid medium plate until the morphology of the bacteria was observed under a microscope. Morphological characteristics of the colonies were observed by optical microscopy and scanning electron microscopy. Gram staining was performed using the Gram Staining Kit (catalog number: G1060; Solarbio, Beijing, China). The V-P, nitrate reduction, and catalase tests were conducted according to the Handbook for the Identification of Common Bacterial Systems [28]. The 16 S rRNA gene was amplified using 27 F and 1492 R primers and sequenced as previously described [29]. Based on the morphology, Gram staining, catalase testing, and 16 S rRNA gene sequencing, the strain was identified as *L. rhamnosus*. The *L. rhamnosus* strain was cultured to 1 × 10^10^ CFU/ml in MRS medium, centrifuged at 4,000 g for 30 min to collect bacterial precipitate, then washed twice and suspended in distilled water, heated at 80℃ for 30 min, and freeze-dried. The determination of effective inactivation was to culture the freeze-dried sample at 37 °C for 48 h without colony formation.

### Study design and measurement of growth indicators

This study was approved by the Biomedical Research Ethics Committee of Hunan Agricultural University (approval number: Lunshenke 2023 No. 127) and was conducted in accordance with its guidelines.

A total of 120 Duroc × Landrace × large hybrid weaned piglets with consistent body weights (8.31 ± 0.16 kg) were purchased from Dayuji Animal Husbandry Technolgy Co., Ltd (Beijing. China) and divided into five groups with six replicates per group and four pigs per replicate based on the principle of similar body weight and the same weight between males and females. Pigs in the control group (CON) were fed a basal diet (Table 1), and those in the ILR1, ILR2, ILR3, and ILR4 groups were fed basal diets supplemented with 0.1, 0.2, 0.3, and 0.4% ILR, respectively.

**Table 1:** Composition and nutrient levels of the basal diet (air-dry basis)

Items	Content (%)		
Ingredients		Calculated value of nutrients (%)	
Corn	56.00	Crude protein	19.00
Soybean mead	12.00	Crude fiber	4.20
Wheat bran	10.00	Lysine	1.35
Extruded soybean	8.00	Threonine	0.76
Fish meal	3.00	Methionine	0.46
Whey powder	3.00	Calcium	0.70
Sucrose	2.00	Total phosphorus	0.65
Zeolite powder	1.00	STTDP	0.35
CaHPO_4_	1.00		
Soybean oil	1.00		
Premix	3.00		
Total	100		

Premix provides 120 mg of Fe, 20 mg of Cu, 100 mg of Zn, 40 mg of Mn, 0.3 mg of Se, 0.5 mg of I, 0.2 mg of Co, 12,000 IU of VA, 2500 IU of VD3, 35 IU of VE, 4 mg of VK3, 1.5 mg of VB1, 4.5 mg of VB2, 3.5 mg of VB6, 0.05 mg of VB12, 50 mg of nicotinic acid, 2 mg of folic acid, 20 mg of pantothenic acid, and 0.4 mg of biotin per kilogram of feed. STTDP: standard total tract digestible phosphorus

A 28-day formal experiment after a 5-day pre-feeding was conducted at the Experimental Base of Chuangzhan Bona Agricultural Technology Co., Ltd. (Zhaoqing, Guangdong, China). During the experiment, the pigs were raised in the same feeding environment and were immunized and sterilized in strict accordance with the management methods of the pig farm. All fences were equipped with automatic feeders and drinking fountains for free access to feed and water.

On the 1st and 28th day of the formal experiment, the initial body weights (IBWs) and final body weights (FBWs) of the weaned piglets were measured, and daily feed consumption and surplus were recorded to calculate the average daily gain (ADG), average daily feed intake (ADFI), and feed conversion ratio (F/G).

At the end of the experiment, six weaned piglets with body weights close to the average of the group were collected from each group, and blood samples were collected from the ear veins after fasting for 6 h. After standing for 30 min, the serum was obtained from blood by centrifugation at 4,000 rpm at 4 °C for 20 min and stored at − 80 °C. After blood collection, the piglets were euthanized in a commercial slaughterhouse (Zhaoqing, Guangzhou, China) by carbon dioxide asphyxiation with less than 2% oxygen (air replaced with carbon dioxide). Intestinal tissue was sampled immediately after dissection, and each intestinal segment was ligated. Colon contents were collected, subsequently transferred to liquid nitrogen for rapid freezing, and then transferred to a − 80℃ refrigerator for storage.

### Determination of serum indicators

Serum total protein (TP), albumin (ALB), aspartate aminotransferase (AST), alanine aminotransferase (ALT), total cholesterol (T-CHO), high-density lipoprotein cholesterol (HDL-C), low-density lipoprotein cholesterol (LDL-C), blood urea nitrogen (BUN), lysozyme, total antioxidant capacity (T-AOC), glutathione peroxidase (GSH-Px), total superoxide dismutase (T-SOD), and malondialdehyde were measured using appropriate kits (Nanjing Jiancheng Bioengineering Institute, Nanjing, Jiangsu, China). IgA, immunoglobulin M (IgM), and immunoglobulin G (IgG) were measured using pig IgA ELISA kits (detection range: 0.146–37.5 µg/ml; catalog number: CSB-E13234p), IgM ELISA kits (detection range: 0.039–10 µg/ml; catalog number: CSB-E06805p), and IgG ELISA kits (detection range: 0.586–150 µg/ml; catalog number: CSB-E06804p), respectively (Cusabio, Wuhan, Hubei, China).

### Microbiota composition analysis of colon contents

Colonic microbiome DNA was extracted using the TGuide S96 kit (TianGen, Beijing, China). The hypervariable V3-V4 region of the 16 S rDNA was amplified using primers 338 F and 806R as previously described with modifications [30]. Briefly, polymerase chain reactions (PCRs) were performed in duplicate with a 25-µl reaction mix containing 1 × PCR buffer, 0.25 U of Taq DNA polymerase (Transgen, Beijing), 0.2 mM of each deoxynucleoside triphosphate, 1.0 µM of each primer, and 10 ng microbial genomic DNA. The thermal cycling procedure consisted of an initial pre-denaturation step at 94 °C for 10 min that was followed by 30 cycles of 94 °C for 30 s, 56 °C for 30 s, and 72 °C for 30 s and a final extension at 72 °C for 10 min. Subsequently, the PCR products were detected using 1.8% agarose gel electrophoresis and purified using an AxyPrep DNA gel extraction kit (Axygen, China). Sequencing was performed using a Sequel II sequencer (PacBio, Silicon Valley, CA, USA) (Biomarker Technologies, Beijing, China) [31]. Raw data were merged using FLASH version 1.2.11 The merged tags were quality controlled using Trimmomatic version 0.33. High-quality tags were obtained after removing chimeric sequences using UCHIME version 8.1 and were clustered into observed taxonomic units (OTUs) with 97% sequence similarity using USEARCH version 10.0. Each feature was annotated using the Silva rRNA database [32]. α-Diversity indices were calculated using Mothur 1.30. β-Diversity analysis was performed using QIIME2 [33] and visualized by component analysis (PCA).

### Non-targeted metabolomes analysis of colon contents

The colon content samples were added to an extraction solution (methanol: acetonitrile: water = 2:2:1, interior label concentration of 2 mg/L) containing an interior label (1000:2), vortexed mixed for 30 s, then ground, and sonicated. After standing at − 20 °C for 1 h, the samples were centrifuged at 1,200 rpm for 15 min at 4 °C. The supernatant was transferred into an EP tube, dried in a vacuum concentrator, and re-dissolved in acetonitrile solution (1:1 acetonitrile: water). Subsequently, the supernatant samples were obtained by vortexing, ultrasonication, and re-centrifugation and then used for subsequent detection.

A non-targeted metabolomics assay was performed by Biomarker Biotechnology Co., LTD (Beijing, China). The LC-MS system consisted of an Acquity I-Class PLUS ultra-high performance liquid chromatography-mass spectrometer (Waters, Framing, Massachusetts, USA) in tandem with a Xevo G2-XS QT of high-resolution mass spectrometer (Waters) with an Acquity UPLC HSS T3 column (1.8 μm 2.1*100 mm; Waters). Raw data were collected using MassLynx V4.2, and peaks were extracted, aligned, and processed using Progenesis QI software. Material identification was carried out using Progenesis QI software with online METLIN, public, and Biomark self-built databases. Theoretical fragment identification was performed simultaneously. The mass deviation of the parent ion was within 100 ppm, and that of the fragment ion was within 50 ppm. The ropls R package was used for orthogonal partial least squares discriminant analysis (OPLS-DA), and permutation was used to test the reliability of the model. The identified metabolites were annotated using the KEGG, HMDB, and LIPID MAPS databases. The variable importance in projection (VIP) of the OPLS-DA model obtained from multivariate analysis was analyzed, and differential metabolites were identified for metabolic pathway analysis, combined with the p-values of univariate analysis.

### Data analysis

Data were analyzed by one-way ANOVA using SPSS 20.0. Tukey’s test was used for multiple comparisons, and data are presented as means ± standard error. Histograms were plotted using the GraphPad Prism 6 software (GraphPad Prism Inc., USA). Spearman analysis was performed to reveal the correlation between gut microbial communities and the altered metabolites, and the R pheatmap package was used for visualization. Results were considered significant at *P* < 0.05.

## Results

### Effect of ILR on the growth performance of weaned piglets

**Fig. 1:**
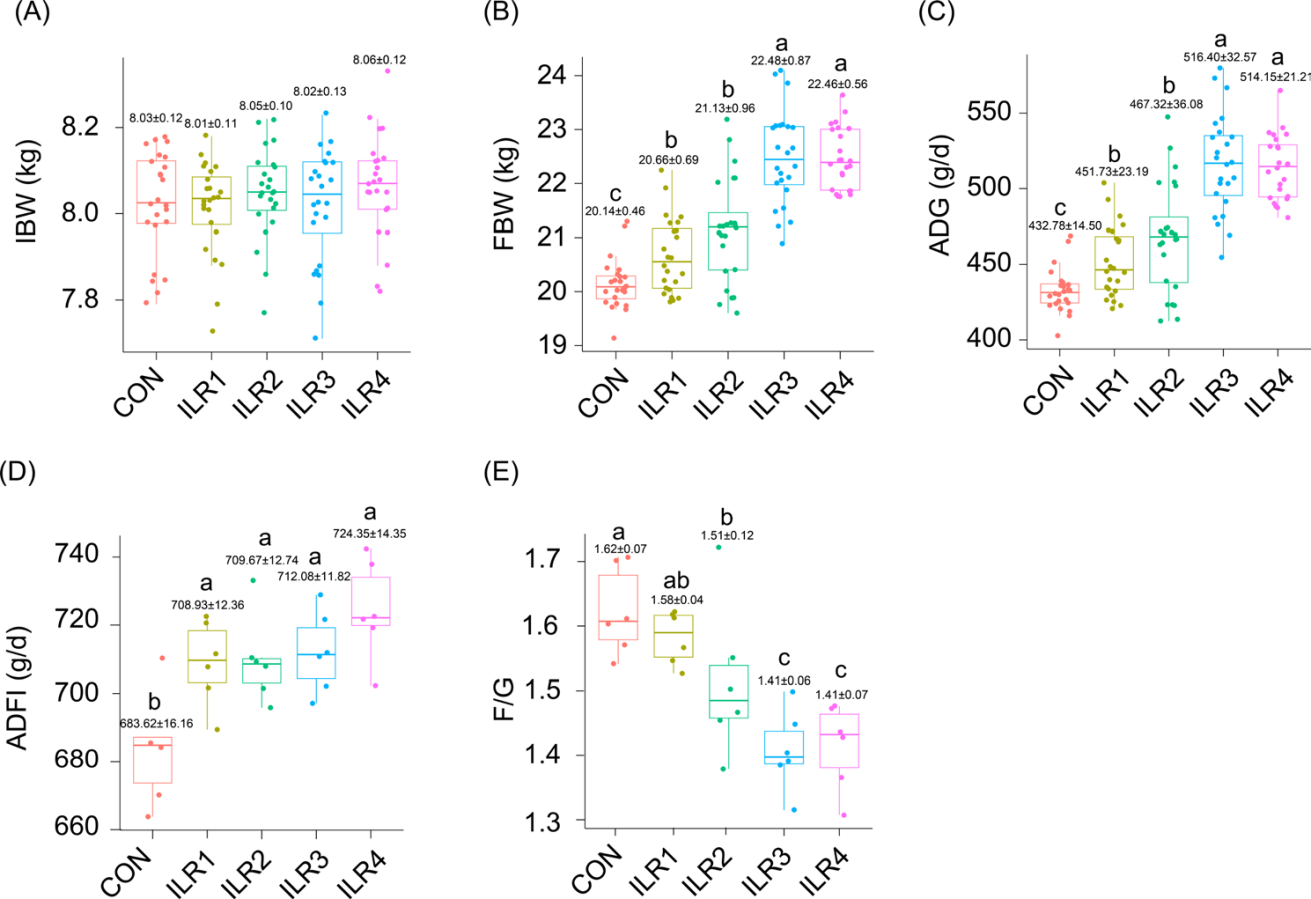
Effect of inactivated *Lactobacillus rhamnosus* on the growth performance of weaned piglets. (**A**) initial body weight (IBM); (**B**) final body weight (FBW); (**C**) average daily gain (ADG); (**D**) average daily feed intake (ADFI); (**E**) feed conversion ratio (F/G). Different lowercase letters above the boxes indicate significant differences between the two datasets

There were no significant differences in IBW among the weaned piglets in any of the groups (*P* > 0.05; Fig. 1A), whereas the FBW of all treatment groups was significantly higher than that of the control group (*P* < 0.05; Fig. 1B). Compared to the control, the ADG of each experimental group significantly increased by 4.38%, 7.98%, 19.32%, and 18.80%, respectively (*P* < 0.05; Fig. 1C), and the F/G decreased by 0.63%, 3.80%, 12.66%, and 10.76%, respectively (Fig. 1E). Although the ADFI in each treatment group was significantly higher than that in the control group, there were no significant differences in ADFI among the treatment groups (Fig. 1D). These results revealed that the ILR3 and LIR4 groups exhibited the best weight gain and feed utilization of weaned piglets, but there was no significant difference between the groups (Fig. 1).

### Effect of ILR on serum biochemical indicators in weaned piglets

**Fig. 2:**
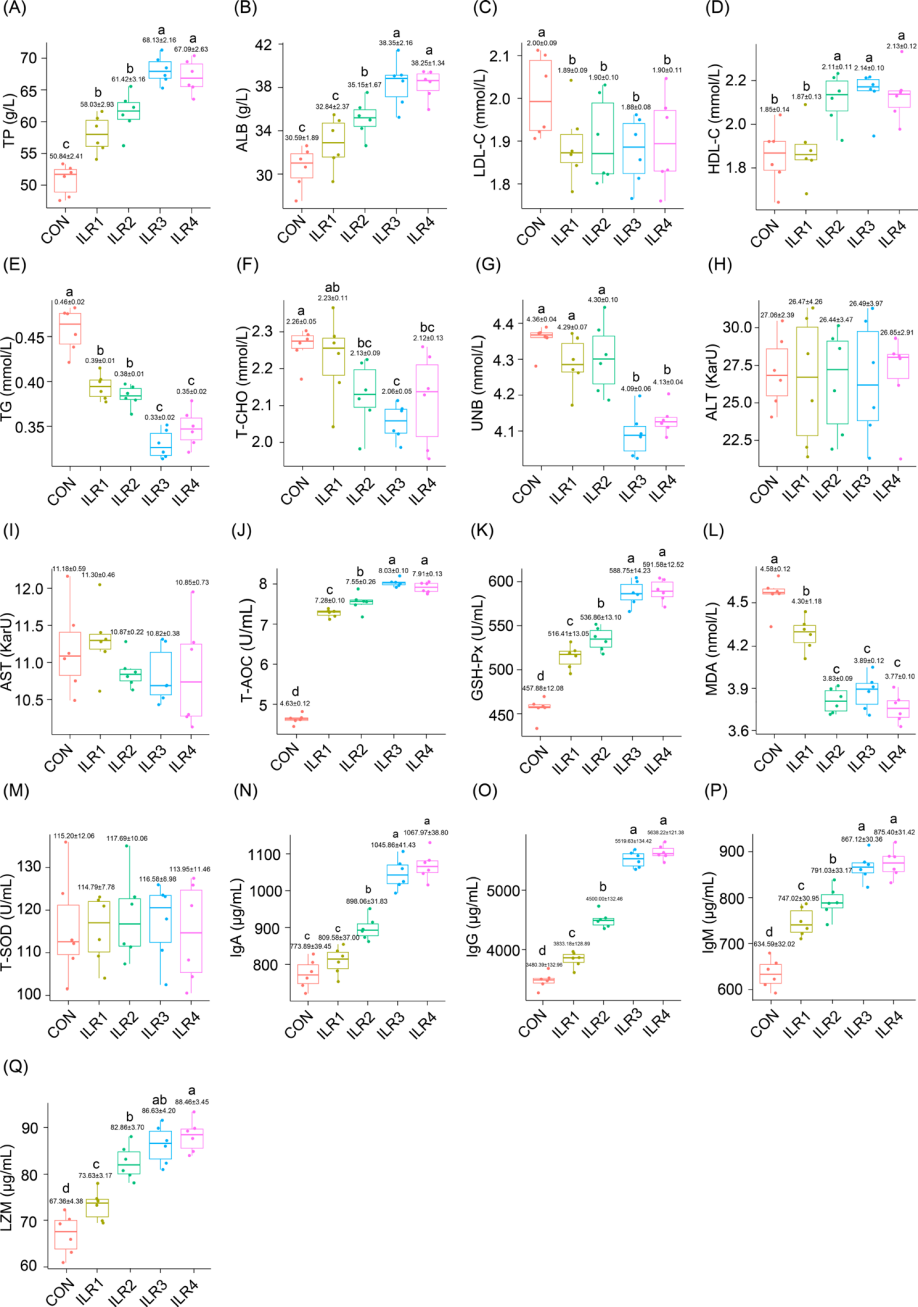
Effect of inactivated *Lactobacillus rhamnosus* on serum biochemical indicators in weaned piglets. (**A**) total protein (TP); (**B**) albumin (ALB); (**C**) low-density lipoprotein cholesterol (LDL-C); (**D**) high-density lipoprotein cholesterol (HDL-C); (**E**) TG; (**F**) total cholesterol (T-CHO); (**G**) blood urea nitrogen (BUN); (**H**) alanine aminotransferase (ALT); (**I**) aspartate aminotransferase (AST); (**J**) total antioxidant capacity (T-AOC); (**K**) glutathione peroxidase (GSH-Px); (**L**) malondialdehyde (MDA); (**M**) total superoxide dismutase (T-SOD); (**N**) immunoglobulin A (IgA); (**O**) immunoglobulin G (IgG); (**P**) immunoglobulin M (IgM); and (**Q**) lysozyme (LZM)

The serum TP of the weaned piglets treated with ILR was significantly higher than that of the control (*P* < 0.05), and the effects of ILR3 and ILR4 were the most obvious, whereas there was no significant difference between these two groups (*P* > 0.05; Fig. 2A). Serum albumin and HDL-C levels in the ILR2, ILR3, and ILR4 groups were significantly higher than those in the control group (*P* < 0.05), while serum LDL-C and triglyceride (TG) levels in each treatment group were significantly lower than those in the control group (*P* < 0.05; Fig. 2C and E). Serum T-CHO levels in the ILR2, ILR3, and ILR4 groups were significantly lower than those in the control group, and those in the ILR3 group decreased the most and were not significantly different from those in the ILR2 and ILR4 groups (Fig. 2F). BUN levels in the ILR3 and ILR4 groups were significantly lower than those in the control group (*P* < 0.05; Fig. 2G). There were no significant differences in serum ALT and AST levels between the treatment and control groups (*P* > 0.05; Fig. 2H and I).

Serum T-AOC levels in the ILR addition group were significantly elevated compared to those in the control group (*P* < 0.05), and the ILR3 group exhibited the highest elevation of 74.08% (Fig. 2J). Serum GSH-Px levels in each treatment group were significantly higher than those in the control group (*P* < 0.05), and those in the ILR3 and ILR4 groups increased by 28.67% and 29.54%, respectively (Fig. 2K). The serum malondialdehyde level decreased significantly compared to that in the control (*P* < 0.05), with the ILR4 group exhibiting the greatest decrease, although there was no significant difference among the ILR2, ILR3, and ILR4 groups (*P* > 0.05; Fig. 2L). T-SOD activity was not significantly different among the groups (*P* > 0.05; Fig. 2M).

The activities of IgA, IgG, IgM, and lysozyme in weaned piglets fed ILR were significantly higher than those in the control (*P* < 0.05) and exhibited an increasing trend with increasing ILR supplementation (Fig. 2N-Q). However, these immune parameters were not significantly different between the ILR3 and ILR4 groups (*P* > 0.05; Fig. 2N-Q).

### Effect of ILR on the colon microbiota structure of weaned piglets

**Fig. 3:**
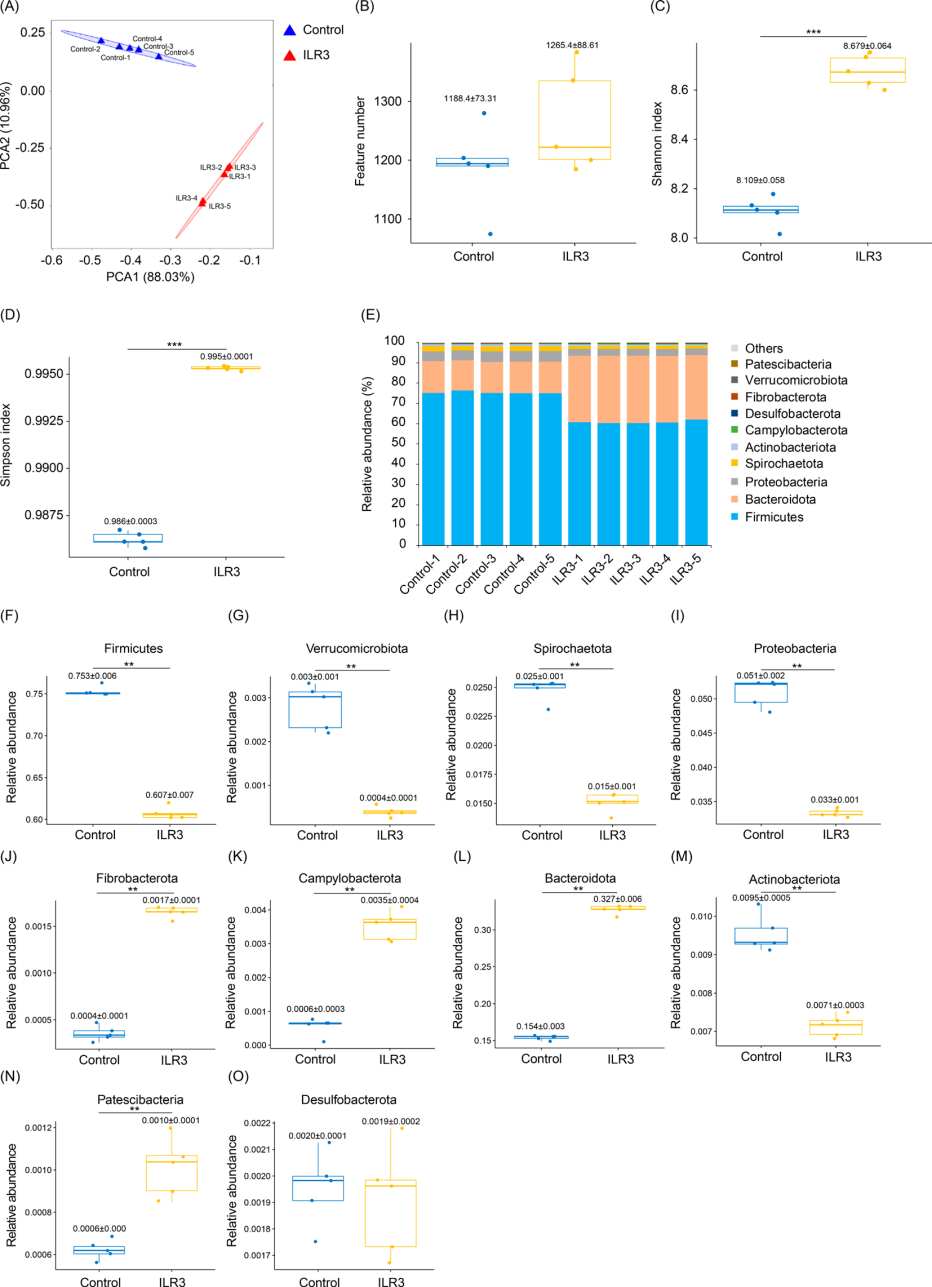
Effect of inactivated *Lactobacillus rhamnosus* on the colon microbiota structure of weaned piglets. (**A**) Principal component analysis profile; (**B**) Feature number; (**C**) Shannon index; (**D**) Simpson index; (**E**) Relative abundances of the top 10 phyla in the colon microbiota of weaned piglets; (**F**) Relative abundance of Firmicutes; (**G**) Relative abundance of Verrucomicrobiota; (**H**) Relative abundance of Spiroochaetota; (**I**) Relative abundance of Proteobacteria; (**J**) Relative abundance oof Fibrobacterota; (**K**) Relative abundance of Campylobacterota; (**L**) Relative abundance of Bacteroidota; (**M**) Relative abundance of Actinobacteriota; (**N**) Relative abundance of Patescibacteria; (**O**) Relative abundance of Desulfobacterota. ** *P* < 0.01; *** *P* < 0.001

Based on the results of growth performance and serum parameters, the control and ILR3 groups were selected to analyze the structure and metabolic profiles of the colonic microbiota in weaned piglets. In total, 4,521 OTUs were identified in both groups. Principal component analysis (PCA) demonstrated clear differences in colonic microbiota between the ILR3 and control groups (Fig. 3A). Although there were no significant differences in OTU number (*P* > 0.05; Fig. 3B), the Shannon and Simpson indices in the ILR3 group were significantly higher than those in the control (*P* < 0.05; Fig. 3C and D), thus indicating that ILR increased the α-diversity of colonic microbiota in weaned piglets.

Bacteroidetes and Firmicutes dominated the colonic microbiota of both groups (Fig. 3E). The relative abundances of Firmicutes, Verrucomicrobiota, Spirochaetota, Proteobacteria, Actinobacteria, and Patescibacteria in the ILR3 group were significantly lower than those in the control group (*P* < 0.05; Fig. 3F-I and M, and 3N), whereas those of Fibrobacterota, Campylobacterota, and Bacteroidetes were significantly higher than those in the control group (*P* < 0.05; Fig. 3J-L).

**Fig. 4:**
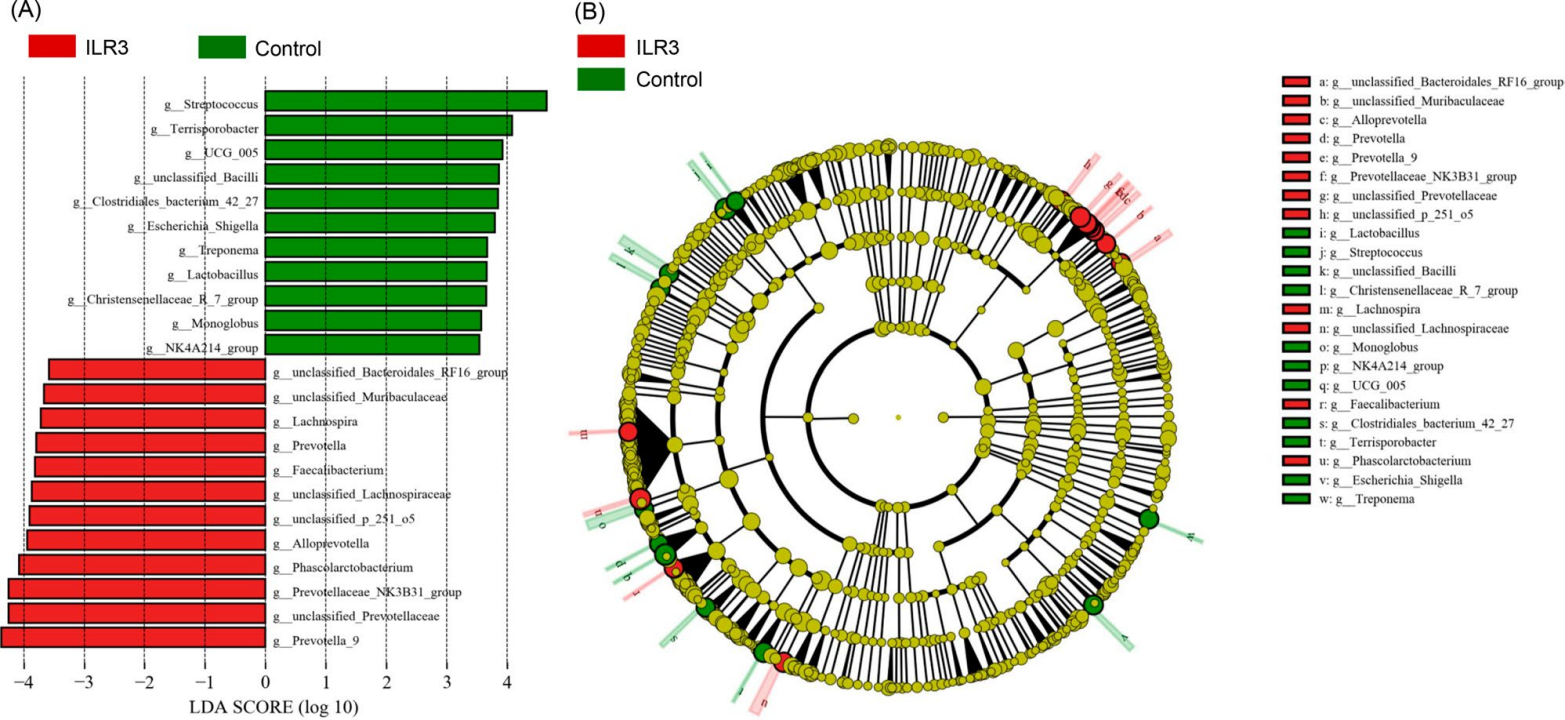
LEfSe results indicated the significantly altered genera of colon microbiota in weaned piglets treated with inactivated *Lactobacillus rhamnosus*

The relative abundances of *Prevotella* and *Alloprevotella* in Prevotellaceae and *Phascolarctobacterium*, *Faecalibacterium*, *Lachnospira*, and many unclassified genera in the ILR3 group were significantly increased compared to the control, and those of *Streptococcus*, *Terrisporobacter*, *Treponema*, *Escherichia_Shigella*, and many unclassified genera were significantly decreased (Fig. 4).

### Effect of ILR on the colon content metabolites in weaned piglets

**Fig. 5:**
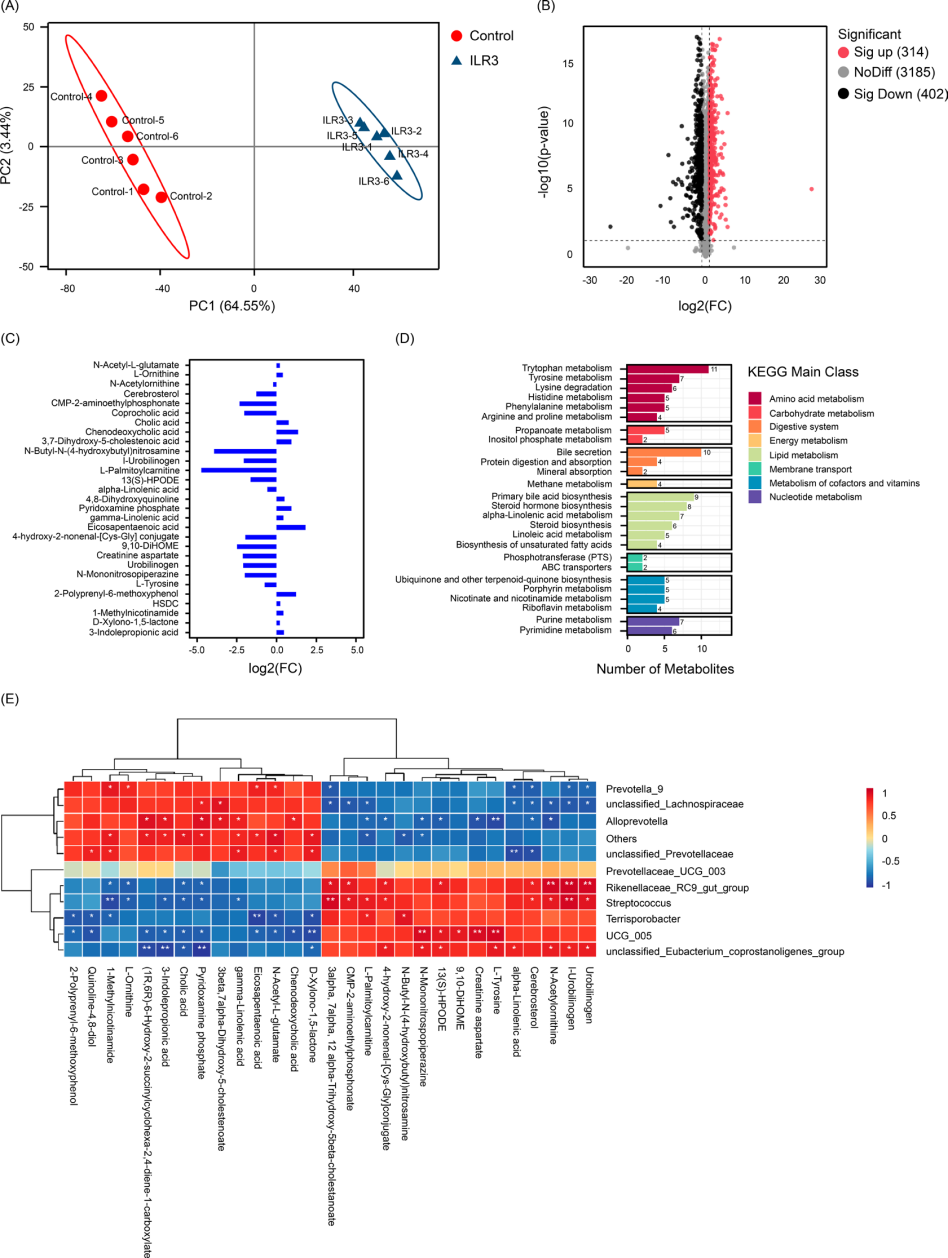
Effect of inactivated *Lactobacillus rhamnosus* on the metabolites of colon contents in weaned piglets. (**A**) OPLS-DA scores of all samples in the default mode; (**B**) Volcano plot indicates differential metabolites of colon contents in weaned piglets treated by inactivated *L. rhamnosus*; (**C**) Main significantly different metabolites of contents in weaned piglets treated with inactivated *L. rhamnosus*; (**D**) Classification diagram indicates the distribution of differential metabolites in KEGG pathways; (**E**) Heatmap indicates Spearman correlations between the main genera of colon microbiota and content metabolites in weaned piglets. * *P* < 0.05; ** *P* < 0.01

The PCA results of the colonic content metabolites demonstrated a clear differentiation between the control and ILR treatments, thus indicating an altered metabolic profile in the colonic contents of weaned piglets (Fig. 5A). Based on a VIP value > 1 in the model and a p-value < 0.05 of univariate analysis, 314 metabolites were significantly upregulated and 402 metabolites were significantly downregulated in the ILR3 group compared to levels in the control group (Fig. 5B). In particular, in the ILR3 group, N-acetyl-L-glutamate (NAG), L-ornithine, cholic acid, chenodeoxycholic acid, 3,7-dihydroxy-5-cholestenoic acid, quinoline-4,8-diol, pyridoxamine phosphate, γ-linolenic acid, eicosapentaenoic acid, 2-polyprenyl-6-methoxyphenol, HSDC, 1-methylnicotinamide, D-xylono-1,5-lactone, and 3-indolepropionic acid were significantly up-regulated, and N-acetylornithine, cerebrosterol, coprocholic acid, 13(S)-HPODE, α-linolenic acid, L-tyrosine, N-butyl-N-(4-hydroxybutyl)nitrosamine, 9, 10-dihome, CMP-2-aminoethylphosphonate, creatinine aspartate, urobilinogen, I-urobilinogen, N-mononitrosopiperazine, and 4-hydroxy-2-nonenal-[Cys-Gly] conjugate were significantly down-regulated compared to levels in the control (Fig. 5C). Furthermore, metabolic pathway enrichment analysis demonstrated that the differences in metabolites between ILR3 and control groups were primarily related to amino acid metabolism, urea cycle, lipid metabolism, cofactor metabolism, and vitamin metabolism pathways (Fig. 5D).

Spearman’s correlation analysis demonstrated that cholic acid was significantly negatively correlated with UCG_005, *Terrisporobacter*, and *Streptococcus* in the primary bile acid biosynthesis pathway (*P* < 0.05; Fig. 5E). Chenodeoxycholic acid was negatively correlated with UCG_005 and positively correlated with *Alloprevotella*. Coprocholic acid and 24OHC were significantly negatively correlated with *Prevotella* 9 and unclassified Lachnospiraceae and significantly positively correlated with *Streptococcus*. Moreover, *Prevotella* 9 was significantly negatively correlated with α-linolenic acid and positively correlated with NAG, eicosapentaenoic acid, L-ornithine, and 1-methylnicotinamide. *Alloprevotella* was significantly negatively correlated with N-mononitrosopiperazine, creatinine aspartate, 4-hydroxy-2-nonenal-[Cys-Gly] conjugate, and L-tyrosine and positively correlated with γ-linolenic acid, pyridoxamine phosphate, 3-indolepropionic acid, and SHCHC. *Streptococcus* was significantly and positively correlated with I-urobilinogen and significantly negatively correlated with 1-methylnicotinamide levels. *Terrisporobacter* was significantly positively correlated with n-butyl N-(4-hydroxybutyl) nitrosamine and negatively correlated with D-xylono-1,5-Lactone, NAG, eicosapentaenoic acid, 1-methylnicotinamide, quinoline-4,8-diol, and 2-polyprenyl-6-methoxyphenol (*P* < 0.05; Fig. 5E).

## Discussion

Inactivated lactic acid bacteria possess excellent application prospects for animal production and are more economical and convenient to use than are live bacteria [24]. Adding heat-inactivated *L. rhamnosus* to the diet of growing pigs reduced diarrhea and improved growth performance [21]. In the present study, our results demonstrated that ILR supplementation significantly improved the growth performance of weaned piglets, and the best effect was achieved when the supplementation level was 0.3%.

Serum antioxidant enzyme activity is an important indicator of health and the ability to remove free radicals [34]. Lactic acid bacteria exert significant immunoregulatory effects, including the activation of macrophages, interferons, and IgA [35]. In this study, supplementation with ILR in the feed of weaned piglets significantly increased serum T-AOC and GSH-Px activity and decreased malondialdehyde levels. Generally, immunoglobulins are directly related to immune function, and lysozymes are involved in innate immunity. This study revealed that supplementation with ILR significantly increased serum IgA, IgG, IgM, and lysozyme levels in weaned piglets, and this is consistent with the effect of live bacteria on weaned piglets [36]. These results indicate that the improvement in the immune function and antioxidant capacity of weaned piglets by *L. rhamnosus* is not caused by its growth in the intestine to stimulate the development of the host intestinal immune system but by its cellular components. The specific cellular components that regulate host immunity and enhance antioxidant capacity require further investigation.

Serum TP, albumin, and BUN levels are related to the growth performance of weaned piglets and are often used as indicators of protein synthesis and metabolism [37]. Previous studies have revealed a strong negative correlation between the biological functions of diet and BUN [38]. In the present study, supplementation with 0.3% and 0.4% ILR significantly increased serum TP and albumin levels and reduced serum BUN levels, indicating that ILR supplementation promoted amino acid utilization and metabolism, and this was beneficial to the health and weight gain of weaned piglets. Serum T-CHO and TG levels reflect changes in the lipolysis rate [39]. The main function of LDL-C is to transport endogenous cholesterol, whereas HDL-C transfers cholesterol from peripheral tissues to the liver for metabolic decomposition [40]. In this study, compared to levels in the control group, serum T-CHO and TG levels in the 0.3% and 0.4% ILR supplementation groups were significantly decreased, whereas HDL-C and LDL-C levels were significantly increased and decreased, respectively, thus indicating that ILR supplementation promotes lipid and cholesterol metabolism in weaned piglets. This was consistent with the results of a previous study examining heat-killed *L. rhamnosus* in mice [41]. The increase in serum AST and ALT levels is likely related to liver injury and is a sensitive marker of hepatocyte damage [42]. Our results revealed that ILR supplementation did not alter serum AST and ALT levels in weaned piglets, thus indicating that ILR supplementation did not damage the liver or myocardial cells of weaned piglets.

In pigs, the gut microbiota is closely related to nutrient metabolism, immune regulation, and defense against pathogens [43]. This study is the first to report that ILR alters the structure and increases the α-diversity of the colonic microbiota in weaned piglets. Previous studies have revealed that increased levels of Firmicutes and decreased levels of Bacteroidetes contribute to obesity and excess body fat [44]. Bacteroidetes contain numerous probiotics and are rich in polysaccharide-degrading enzymes [45]. The ADG and serum levels of TP and BUN suggest that dietary ILR supplementation may contribute to growth and body fat reduction in weaned piglets, and this is consistent with the speculation of He et al. [46]. Proteobacteria are the microbial characteristics of intestinal inflammation [47]. Studies have also demonstrated that an increase in spirochetal levels may cause an inflammatory response in the body, ultimately leading to chronic gastritis in pigs and thereby affecting the digestive system [48]. Our results revealed that ILR supplementation significantly reduced the relative abundances of Proteobacteria and Spirochetes, thus indicating that ILR supplementation is beneficial to host health. *Prevotella* positively correlated with feed efficiency and growth performance [49]. This is likely due to the ability of *Prevotella* to ferment complex dietary polysaccharides [50], thereby promoting the uptake of monosaccharides by the host and conferring a growth performance advantage [51]. Simultaneously, large amounts of short-chain fatty acids (SCFA) are produced [52]. Our results indicate that ILR supplementation significantly increased the relative abundance of *Prevotella* in the colonic microbiota of weaned piglets. Furthermore, the addition of 0.3% ILR significantly increased the abundance of *Phascolarctobacterium* that can colonize the human gut, produce SCFA, and play a beneficial role [53]. SCFA are considered mediators of communication between the gut microbiota and the immune system and help to maintain an anti-inflammatory and pro-inflammatory balance [54]. An increase in an unclassified Lachnospiraceae bacterium also plays an important role in cellulose digestion [55]. The common diet used in this study contained certain complex dietary polysaccharides, and changes in the colonic microbiota likely improved digestion and absorption in growing pigs. This is likely one reason for the significant increase in the feed-to-gain ratio. Gut microbiota enriched in *Prevotella* also reduce cholesterol levels [56]. The reduction in serum cholesterol levels observed in the present study may be related to the high abundance of *Prevotella*. The most significant increase in relative abundance was *Prevotella* 9 that accounted for 5.22% in the ILR3 group and only 0.50% in the control group. Hung et al. [57] demonstrated that the abundance of *Prevotella* 9 in the feces of weaned piglets was negatively correlated with diarrhea and positively correlated with growth performance, whereas a decreased abundance of UCG-005 was positively correlated with diarrhea and negatively correlated with growth performance, and this is consistent with the results of this study. *Alloprevotella* can regulate intestinal inflammation and exhibit anti-inflammatory effects [58], and its relative abundance is inversely correlated with inflammation [59]. Prevotellaceae_NK3B31_group effectively alleviates intestinal inflammation, promotes intestinal nutrient absorption, and reduces immune rejection in autoimmune diseases [60]. *Lachnospira* can produce butyrate, and its low abundance is associated with constipation [61]. *Faecalibacterium* is one of the most common genera in the gut microbiota of healthy adults [62]. It can produce butyrate [63], peptides [64], and extracellular polymeric matrix [65], and other metabolites have been demonstrated to exhibit anti-inflammatory activities in animal models and in vitro experiments [66]. Our results revealed that the abundance of these bacteria increased after ILR supplementation, and this was related to the improvement in antioxidant and immune indices, thus indicating that ILR improve the intestinal microbiota of weaned piglets and enhance immunity.

*Streptococcus* is a major swine pathogen that leads to high economic losses in the pig industry and causes zoonotic infections such as meningitis and sepsis [67]. Therefore, effective treatment and prevention of *Streptococcus suis* infections are important in the pig industry. In this study, ILR supplementation significantly decreased *Streptococcus* numbers, thus indicating that ILR possess good disease-resistant potential. Moreover, the relative abundance of *Terrisporobacter* was significantly decreased in the ILR3 group. *Terrisporobacter* induces postoperative infection in patients [68], and its increased abundance may contribute to increased inflammation and oxidative stress [69]. Other bacteria prone to negative effects have been observed in reduced abundance such as *Treponema*, Christensenelleaceae R-7, and *Escherichia-Shigella*. The Christensenelleaceae R-7 group exhibited a high abundance of bacterial infections, ultimately resulting in loss of appetite and emotional anxiety that were negatively correlated with weight gain [70, 71]. *Escherichia* contains many pathological forms that cause diarrhea, dysentery, and parenteral infections, including urinary tract infections and meningitis [72]. *Shigella* is a major pathogen of bacillary dysentery worldwide and is commonly observed in cholera diarrhea or enterotoxigenic *E. coli* diarrhea [73]. *Escherichia-Shigella* is associated with significant dysregulation of the gut microbiota in patients with tuberculous meningitis [74]. Therefore, ILR optimizes the intestinal microbiota structure of weaned piglets and inhibits pathogenic microorganisms, thereby improving growth performance and immune function, and it possesses good prospects for the development of feed additives.

An important means by which gut microbes influence host health is through metabolites [75]. NAG induces essential allosteric activation of carbamyl phosphate synthetase I (CPS1), a key enzyme in the mammalian urea cycle [76]. NAG deficiency leads to urea cycle disease and carbamyl phosphate synthetase deficiency (CPS1D) [77]. L-ornithine is one of the products in the process of producing urea from the effect of L-arginine on L-arginine. It is a core part of the urea cycle and can remove excess nitrogen [78], promote lipid metabolism, activate the urea cycle, and stimulate urea synthesis [79, 80]. The increase in NAG and L-ornithine in this study suggests that ILR may promote the urea cycle and protein metabolism, maintain nitrogen equilibrium, and maintain health and nutritional status within the body. 1-Methylnicotinamide (MNA), a metabolite of vitamin B3, increases NO release from vascular endothelial cells and lowers blood pressure [81]. It also exerts antithrombotic and anti-inflammatory effects [82]. The increase in MNA observed in the present study may be related to positive changes in serum immune markers in the treatment group.

In the primary bile acid biosynthesis pathway, cerebrosterol and its intermediate product coprocholic acid were decreased in the ILR3 group, whereas its final products cholic acid and chenodeoxycholic acid were increased. Cerebrosterol is highly expressed in diseases such as Alzheimer’s disease and meningitis [83, 84]. A decrease in cerebrosterol levels indicates that more cholesterol is used to synthesize bile acids (BA). Cholic acid (CA) and chenodeoxycholic acid (CDCA) are the primary bile acids synthesized in the liver [85, 86]. BA plays an important role in lipid digestion and absorption and is an important regulator of the intestinal microbiota that is closely related to intestinal microbes and host health [87]. Therefore, ILR may promote fat digestion and absorption, regulate sterol metabolism, and improve feed efficiency by promoting CA and CDCA synthesis through the primary bile acid biosynthesis pathway. α-Linolenic acid (ALA) is an essential fatty acid belonging to the ω-3 series of polyunsaturated fatty acids (PUFAs). ALA can be metabolized into bioactive long-chain PUFAs such as eicosapentaenoic acid (EPA) in the human body [88]. However, EPA is not readily converted from the precursor ALA in humans and other mammals, as the enzyme activity involved in the conversion is weak [89]. In this study, ALA levels decreased and EPA levels increased in the ILR3 group, indicating that ILR can improve related enzyme activities and promote ALA metabolism, although more experimental evidence is needed. γ-Linolenic acid, an anti-inflammatory ω-6 PUFA [90], was increased in the ILR3 group. ω-6 and ω-3 PUFAs play key roles in a variety of biological functions and are essential for health [91]. Fermented rhamnose milk can participate in α-linolenic acid metabolism and arachidonic acid metabolism, promote fatty acid degradation, and regulate lipid metabolic homeostasis in rats [92]. This is consistent with our results with the addition of ILR.

Correlation analysis demonstrated that *Terrisporobacter* was significantly negatively correlated with ω-3 PUFA eicosapentaenoic acid. A previous study revealed that *Terrisporobacter* was significantly positively correlated with TC, TG, and LDL-C and negatively correlated with HDL-C and that reducing *Terrisporobacter* may increase the levels of unsaturated fatty acids and BAs [69]. Guo et al. [93] speculated that *Terrisporobacter* may be involved in the regulation of enzymes involved in BA metabolism or lipid biosynthesis, ultimately leading to higher lipid levels and dyslipidemia. Our results are consistent with these findings. Moreover, certain bioactive substances such as MMA and pyridoxamine phosphate (PMP) were upregulated in the ILR3 group. MMA also exerts anti-inflammatory effects [82]. PMP is a bioactive vitamin B6 [94]. Vitamin B6 exerts a positive effect on the development of immune organs, serum immunoglobulin content, and growth performance of weanling rabbits [95]. However, they are negatively correlated with *Streptococcus* and positively correlated with an unclassified genus (Prevotellaceae). Moreover, the toxic leukotoxin diol 9,10-dihome [96] was reduced by the addition of ILR and positively correlated with UCG_005. Overall, alterations in gut microbial composition and diversity produced by ILR supplementation may play an important role in the development of gut metabolism, ultimately benefiting the health of weaned piglets. Notably, due to limited experimental funds, we only compared the microbiota and metabolite compositions in the colon contents between the ILR3 and control groups. It is necessary to systematically study the effects of different concentrations of ILR on the composition of microbiota and metabolites in piglet colon contents.

## Conclusions

Dietary supplementation with ILR can effectively improve the growth performance, serum antioxidant and immune indices, and composition and metabolic characteristics of the colonic microbiota of weaned piglets, and the 0.3% supplementation level was the best. The addition of 0.3% ILR increased the α-diversity and optimized the structure and metabolism of colonic microbiota, and this primarily affected amino acid metabolism, urea cycle, lipid metabolism, cofactor metabolism, and vitamin metabolism pathways. Therefore, the results of this study highlight the beneficial effects of ILR on the growth and health of weaned piglets and reveal the potential mechanism by which ILR improves the intestinal system.

## Data Availability

The raw sequences were deposited in the NCBI Sequence Read Archive database with accession number PRJNA1044105.
